# Guiding Molecularly Imprinted Polymer Design by Pharmacophore Modeling

**DOI:** 10.3390/molecules26165101

**Published:** 2021-08-23

**Authors:** Wiebke Derz, Melita Fleischmann, Paul W. Elsinghorst

**Affiliations:** 1 Central Institute of the Bundeswehr Medical Service Munich, 85748 Garching, Germany; wiebkederz@bundeswehr.org (W.D.); melitafleischmann@bundeswehr.org (M.F.); 2 Institute of Nutrition and Food Sciences, University of Bonn, 53115 Bonn, Germany; 3 Food Technology & Nutrition, Management Center Innsbruck, 6020 Innsbruck, Austria; 4 Pharmaceutical Chemistry I, Pharmaceutical Institute, University of Bonn, 53121 Bonn, Germany

**Keywords:** mycotoxins, citrinin, core-shell polymers, molecularly imprinted polymers, MIP, MISPE, pharmacophore modeling

## Abstract

Molecularly imprinted polymers (MIP) combine the selectivity of immunoaffinity chromatography with the robustness of common solid-phase extraction in what is referred to as molecularly imprinted solid-phase extraction (MISPE). This contribution shows how MIP design may be guided by pharmacophore modeling for the example of citrinin, which is an emerging mycotoxin from cereals. The obtained pharmacophore model allowed searching public databases for a set of citrinin-mimicking molecular surrogates. Imprinted and non-imprinted polymers were subsequently obtained through bulk and core-shell polymerization in the presence of these surrogates. Evaluation of their binding ability for citrinin and structurally related ochratoxin A revealed a promising MIP derived from rhodizonic acid. A protocol for MISPE of citrinin from cereals was subsequently developed and compared to immunoaffinity chromatography with respect to clean-up efficiency and recovery.

## 1. Introduction

Throughout the last 20 years, molecularly imprinted polymers (MIP) have gained increased interest in separation science and sample preparation [1,2,3]. Especially where sample preparation relies on sensitive and selective immunoaffinity chromatography (IAC), MIP appear as an attractive alternative, as they can show comparable extraction efficacy while allowing more robust handling. They do not require specific storage conditions, shelf-life is in principle unrestricted, and washing with organic solvents is also possible [2,4]. As such, mycotoxin analysis is one emerging field, where IAC is gradually replaced or accompanied by molecularly imprinted solid-phase extraction (MISPE) [4]. In addition, MISPE cartridges are less cost-intensive and provide independence of commercial suppliers if produced in-house at laboratory scale. However, obtaining comparable selectivity and sensitivity in analyte binding requires the selection of a suitable surrogate template for molecular imprinting, which in the past has usually been based on visual structure comparison by individual researchers. To increase the explorable chemical space beyond this biased perspective, we suggest applying a more general approach based on pharmacophore modeling known from pharmaceutical drug discovery.

Lead identification as an early step in modern drug discovery requires the screening of large libraries of possible candidate molecules for possible ligand-receptor interaction. To reduce costs of in vitro high-throughput screening, in silico pharmacophore modeling is often applied [5]. As drug discovery programs mostly address known targets, receptor-based modeling is commonly applied, where the target receptor is constructed in silico and screened against public databases to identify possibly binding molecules. In case that no information on the target structure is available, but at least one ligand is known, database screening can be carried out using a three-dimensional map (pharmacophore) of possible ligand interactions.

**Figure 1:**
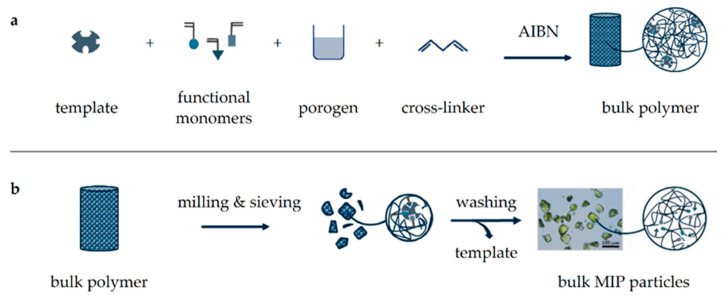
Schematic illustration of MIP synthesis by bulk polymerization. First, a template, functional monomers, cross-linker, and radical starter (AIBN) are dissolved in porogen followed by heat-induced polymerization (**a**). The resulting polymer block is crushed, milled, and sieved to obtain uniformly sized particles, which are washed thoroughly to remove the template (**b**).

Citrinin (CIT) is a polyketide mycotoxin produced by *Aspergillus*, *Monascus*, and *Penicillium* species on vegetable foodstuff, especially on rice and cereal products. Dietary supplements derived from rice fermented by *Monascus purpureus* for the red pigment monacolin K (lovastatin) contain considerable amounts of CIT [6]. While acute toxicity is low, long-term nephrotoxic, hepatotoxic, and cytotoxic effects depending on the frequency and duration of CIT exposure must be taken into account [7,8]. In addition, CIT often co-occurs with other mycotoxins, showing potentially harmful synergism, and a possible carcinogenic effect cannot be excluded [9,10]. EU legislation has set a maximum allowed limit of 2 mg/kg for dietary supplements, and the EFSA recommends a whole-diet TDI of 0.2 µg/kg body weight to avoid nephrotoxic effects [10,11], which is why CIT exposure should be kept as low as possible and needs to be monitored. Analysis of CIT is commonly carried out by LC-FLD and requires thorough sample clean-up usually achieved by IAC or MISPE [12,13,14,15,16], which both follow a similar workflow, where CIT is bound either by specific antibodies (IAC) or in microcavities using comparable molecular interactions (MISPE). Recently, CIT-selective MIP have also been applied for online MISPE as well as for the development of CIT-selective sensors [17,18,19,20,21]. To prepare the necessary MIP, different methods have been developed, the most simple being conventional bulk polymerization (Figure 1) [22,23,24].

**Figure 2:**
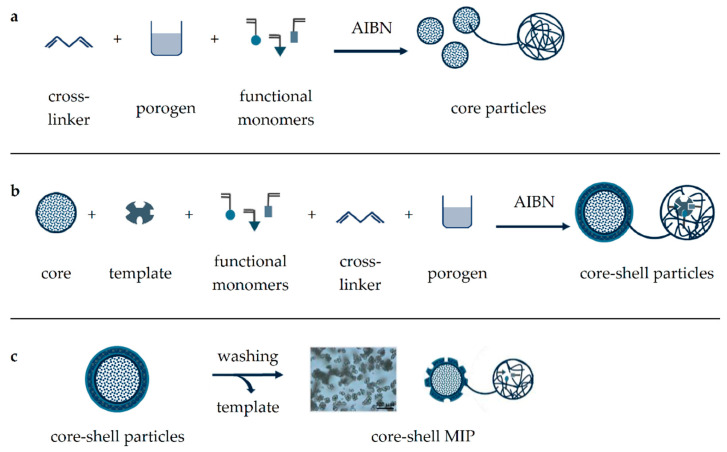
Schematic illustration of core-shell MIP synthesis. Core particles are obtained through heat-induced polymerization of functional monomers using a cross-linker (**a**), this step can be repeated to increase core size. Then, core particles are mixed with the template and functional monomers for MIP-shell formation (**b**), which is released by thorough washing (**c**).

During polymerization, molecular imprinting itself results from a pre-polymerization complex, which is formed by non-covalent interactions between functional monomers and the selected template [25]. Understanding and optimizing the formation of this pre-polymerization complex has often been the result of experimental but also of computational studies [26,27]. The dominating kind of interaction (polar, non-polar, or ionic) can be controlled by the organic solvent added as the porogen. Then, subsequent polymerization by cross-linking results in a solid polymer block, which is crushed, ground, and sieved to obtain particles of defined size. Final thorough washing of these particles removes the template from their microcavities to obtain the respective MIP. Another yet more elaborate way of MIP formation is by precipitation polymerization, which can provide core-shell particles by repeated application and offers several advantages including the elimination of laborious milling and sieving steps, improved template removal due to a thinner MIP layer, and more uniform polymer particles, providing better accessibility of microcavities and binding sites [28,29]. In general, the core-shell polymerization process consists of three main steps: core synthesis, core enlargement, and shell formation (Figure 2). First, microspherical core particles are obtained by polymerization in absence of the template, which in contrast to bulk polymerization requires a larger porogen addition to dilute the polymerization mixture [30,31]. This step can subsequently be repeated to enlarge the core particles before a molecularly imprinted layer is finally introduced and the template removed by washing to provide the core-shell MIP particles.

**Figure 3:**
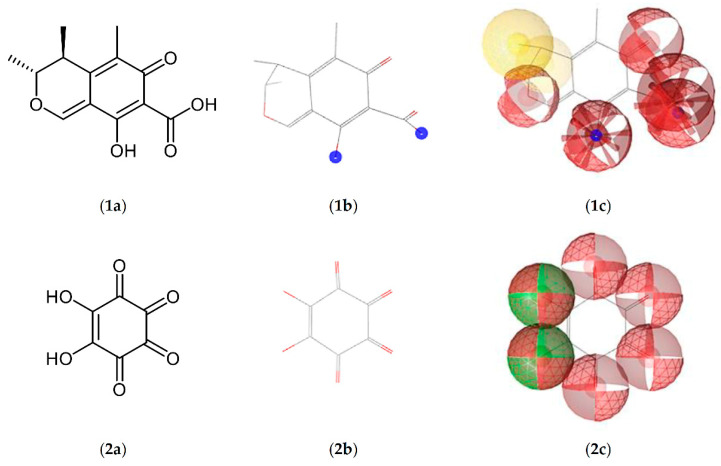
Molecular structures (**a**), results of MMFF94 energy minimization (**b**), and pharmacophore models (**c**) of CIT (**1**) and the surrogate template rhodizonic acid (RHO, **2**). Hydrophobic areas are marked yellow, hydrophilic ones are marked red. Areas enabling hydrogen bonding are marked green, while blue regions denote potential ionic interactions.

Although molecular imprinting by the target analyte itself provides the most specific binding, carry over by polymer bleeding because of non-exhaustive washing will impair (ultra)trace analysis [15]. Instead, a surrogate template must be used, i.e., a molecule that mimics the analytes’ molecular interactions and their spatial orientation, but it does not interfere with the following analysis [22]. Literature reports show that surrogate selection for CIT was predominantly driven by visual structure comparison, which is why mainly naphthoic acid derivatives have been applied [14,15,16,17,18]. To overcome this limited and sometimes biased perspective, computational methods such as pharmacophore modeling may offer additional insight. As depicted in Figure 3, pharmacophore modeling provides a three-dimensional map of the possible molecular interactions of the analyte using categories such as hydrogen bonding, hydrophilic, lipophilic, or ionic interactions. Then, complex matching algorithms use this interaction map (pharmacophore) to search and rank potential surrogates from public databases. The results from this search often belong to completely different compound classes that would have been missed by classic structural analogy.

As a proof of concept, we derived a pharmacophore model of citrinin in search for structurally unrelated (non-naphthoic) surrogate templates and used these for subsequent MIP and MISPE development originating from a CIT-optimized literature protocol [14].

## 2. Results and Discussion

### 2.1. Bulk Polymers

Molecular interactions described by a pharmacophore model are subject to their chemical environment, which in the case of MIP synthesis is mainly governed by the added porogen. As such, a non-polar porogen will limit non-polar interactions while emphasizing polar interactions between surrogate templates and functional monomers and vice versa [23]. Therefore, virtual screening for potential surrogates by pharmacophore modeling must consider the appropriate porogen and be set up correctly. Considering acetonitrile, polar interactions were expected to contribute stronger to polymer binding site formation than non-polar interactions, which is why we focused our pharmacophore search on polar interactions. Using common software tools (PharmIt Search Engine [32], LigandScout [33]; see Materials and Methods for details), a list of possible surrogates was obtained, which were ranked by a match factor (pharmacophore fit score) and further filtered according to commercial availability, reasonable costs, and potential health hazards. From the top of this list, seven easy to handle, low-cost, and non-toxic surrogates were selected and applied for polymer imprinting (Table 1).

MIP obtained by bulk polymerization using these surrogates were subsequently analyzed by LC-FLD for their clean-up efficiency compared to NIP (non-imprinted mock polymers), IAC, and common C_18_ SPE-columns (see Section 4 for details). Polymers imprinted by rhodizonic acid (RHO) appeared most promising with matrix recoveries around 100% comparable to IAC and superior to SPE (Table A1 (Appendix A), 94.0–127.4% vs. 39.4–72.8%) with notable differences between RHO-derived MIP and NIP (94.0–127.4% vs. 21.1–126.5%). Although these initial recoveries were already satisfying, they slightly exceeded 100% in four out of five cases, indicating some remaining matrix interference. Consequently, MISPE washing was optimized to remove these matrix components effectively (see Supplementary Materials for details). Limits of detection (LOD) and quantification (LOQ) obtained after improved MISPE varied somewhat with respect to the matrix investigated except for oats, which were found to be significantly below the current maximum allowed level of 2 mg/kg for dietary supplements [11] (Table 2).

Recovery (55.0–96.5%) and precision (1.9–20.4%) data obtained at LOQ (Table 3, MISPE 1) were also acceptable and in good agreement with previous literature reports for corn (LOD/LOQ: 0.01/0.03 mg/kg, recovery/precision: 87.1 ± 7.2%, [14]). Lower recoveries as observed in wheat products such as pasta (55.0 ± 13.3%) may be a result of the in situ degradation of CIT, which has been shown to decompose faster in these products [35,36]. The unexpected low recoveries obtained by IAC at LOQ may indicate superior performance of the MIP, but IAC precision was very satisfying at higher CIT concentrations (Table A1). The reusability of MISPE cartridges could be shown in the oat matrix at a CIT level of 0.5 mg/kg for at least three cycles with very good recovery (89.3–96.5%) and precision (0.9–1.9%), while at lower concentrations, a memory effect occurred (pasta, wheat flour, and rice products). The impact of this effect is even more significant with a CIT LOQ as low as in the pasta matrix, where MISPE cartridges cannot be reused.

### 2.2. Core-Shell Polymers

Although RHO-derived MIP obtained by bulk polymerization worked pretty well for CIT clean-up, grinding and washing was rather laborious and accompanied by an unavoidable loss of MIP. Therefore, we aimed to improve the manufacturing process by switching to precipitation polymerization for the production of core-shell MIP. Unfortunately, the previously used bulk polymerization mixture proved unsuitable for the production of core-shell particles, but after several attempts, we obtained RHO-imprinted core-shell particles suitable for MISPE using a methanol/acetone mixture as the porogen, ethylene glycol dimethacrylate (EGDMA) as the cross-linker, and *N*,*N*-dimethylaminoethyl methacrylate (DAEM) as the functional monomer (see Supplementary Materials for details). For comparison, also, bulk MIP and NIP comparable to these core-shell polymers were prepared using EGDMA as the cross-linker and methanol as the porogen (type II). Core-shell as well as bulk MIP and NIP were subsequently evaluated for their CIT recovery efficiency from methanol/water (70/30, *v*/*v*) as well as from oats (Table 4).

Surprisingly, recovery (90.0–105.5%) and precision (0.3–2.9%) data were very encouraging for both type II bulk and core-shell polymers, with the LOD and LOQ of core-shell MIP and NIP resembling those previously obtained for bulk MIP. Again, recovery rates at LOQ reached very satisfying 85.1–97.9% with a precision of 1.1–3.4%, regardless of imprinting (Table 5). Since imprinting by RHO is mainly driven by polar interactions, replacing aprotic acetonitrile by more polar and protic methanol may have limited the necessary interactions between the functional monomers and RHO. To confirm this hypothesis, core-shell MIPs and NIPs were also prepared using acetonitrile as the porogen. The resulting particles were much finer than the ones obtained with methanol, and the polymer was very voluminous with a fluffy texture. Subsequent MISPE revealed an 89.2 ± 1.3% to 74.5 ± 2.8% difference between these MIP and NIP, supporting the hypothesis of imprinting suppression by porogen switching from acetonitrile to methanol.

### 2.3. Clean-Up of Ochratoxin A

**Figure 4:**
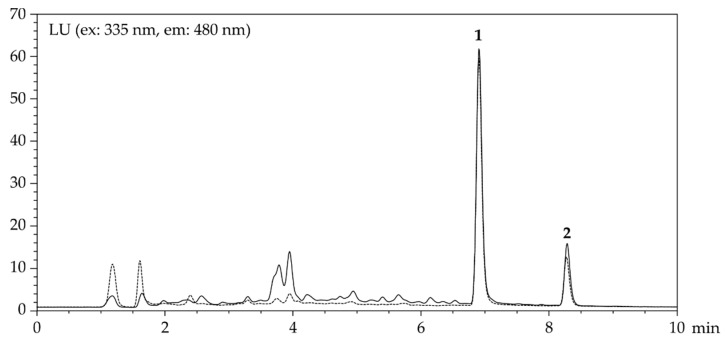
Chromatograms of CIT (**1**, 400 ng/mL) and OTA (**2**, 30 ng/mL) of oats spiked before (—) and after (---) MISPE (see Materials and Methods for details).

The structural similarity to CIT suggested that the prepared core-shell polymers might also be able to bind ochratoxin A (OTA). Notably, MISPE of OTA using methanol/water (70/30, *v*/*v*) as well as oat extracts revealed very acceptable recovery and precision data with slightly better performance of NIP (Table 6). Recoveries obtained in combination experiments (CIT: 89.3 ± 4.0%, OTA: 89.0 ± 2.7%) were very satisfying and demonstrate the applicability of these core-shell NIPs for the simultaneous clean-up of these two mycotoxins CIT and OTA (Figure 4).

## 3. Conclusions

The identification of suitable surrogate templates plays a central role in MIP design for trace analysis. While MIP design strategies optimizing the pre-polymerization complex (monomer mixture composition) have been aided by computational simulations before [27,37], pharmacophore modeling goes one step ahead, mapping the analytes’ interaction potential to discover possible surrogates from chemical space that would have been missed by traditional methods. Even when starting from an already optimized MIP production process, a pharmacophore-based surrogate search can be applied to follow-up improvement studies. Subsequent in-house MIP production offers the selectivity usually associated with IAC at the affordable price of common SPE with independence from commercial suppliers or specific storage conditions. The bulk polymers reported here for CIT clean-up are shelf-stable while offering robust handling. When switching from bulk to core-shell MIP production, careful attention must be paid to a possible change in porogen. Changing polarities or protic/aprotic conditions will affect the pharmacophore search, as molecular interactions can be susceptible to even slight modifications. By chance, an additional NIP was discovered that is capable of simultaneously recovering CIT and OTA from oats and common extract solvents.

## 4. Materials and Methods

### 4.1. Reagents and Materials

Citrinin (CIT), 1-hydroxy-2-naphthoic acid, 1,4-dihydroxy-2-naphthoic acid, 2,2-azobis isobutyronitrile (AIBN), trimethylolpropane trimethacrylate (TRIM), methacrylic acid (MAA), and PBS tablets were obtained from Sigma (Steinheim, Germany). Barium chloride, *N*,*N*-dimethylaminoethyl methacrylate (DAEM), ethylene glycol dimethacrylate (EGDMA), methacrylamide (MAM), salicylic acid, acetic acid, ammonia (25% aq.), sodium hydroxide solution (1M), orthophosphoric acid, and Tween 20 were purchased from Merck (Darmstadt, Germany). LC-grade acetonitrile and methanol were from VWR (Langenfeld, Germany), acetone was from Honeywell (Seelze, Germany). 2-Naphthoic acid was obtained from Acros Organics (Geel, Belgium), 2-oxocyclohexane carboxylic acid was obtained from Fluorochem (Karlsruhe, Germany), and rhodizonic acid dehydrate (RHO) was obtained from abcr (Karlsruhe, Germany). Ochratoxin A (OTA) was provided by HPC Standards (Borsdorf, Germany) and EASI-EXTRACT^®^ CITRININ columns were provided by R-Biopharm Rhone Ltd. (Glasgow, UK). Cereal products (wheat flour, pasta, rice, whole grain rice crispies, and oat flakes) were purchased from local markets and did not contain detectable levels of CIT. CIT stock and working solution were prepared according to DIN EN 17203 [38] and stored in acid-washed amber vials at −20 °C.

### 4.2. Pharmacophore Modeling

Briefly, a three-dimensional structure file (SDF) of CIT was downloaded from PubChem (CID: 54680783) and submitted to the PharmIt Search Engine [32] to generate a corresponding pharmacophore model. PharmIt independently identifies possible interactions and classifies them as hydrophobic or polar/ionic (further features can be added manually if necessary). In case of CIT, five hydrogen acceptors, one hydrogen donor, which is at the same time an anionic interaction site, and four hydrophobic areas were detected. Considering the applied porogen, formation of the pre-polymerization complex was expected to be mainly driven by polar and ionic interactions. Hydrophobic interactions were consequently considered irrelevant and excluded manually before a pharmacophore search was carried out accessing public databases (PubChem, ZINCPharmer) for possible surrogates [39]. Results from this screening were subsequently downloaded and joined into a local database using LigandScout [33], which provides MMFF94 energy minimization of input molecules generating reasonable conformers of each hit. By comparing this local database with an MMFF94 energy-minimized pharmacophore model of CIT, results were ranked according to the pharmacophore fit score offered by LigandScout. Best matching, commercially available pharmacophores were subsequently used for polymer imprinting.

### 4.3. Bulk Polymer Synthesis

MIP were prepared by thermoinitiated bulk polymerization according to Appell et al. [14]. Each batch consisted of two headspace vials (20 mL) that were charged with RHO (200 mg, 1 mmol) or, when comparing previously identified surrogates, the respective surrogate template (1 mmol), acetone (1.875 mL), acetonitrile (5.625 mL), and DAEM (0.675 mL, 4 mmol). The vials were vortexed, sealed, and sonicated for 15 min before storage at 4 °C for 4 h. Next, TRIM (4.79 mL, 15 mmol) was added, and the mixture was flushed with nitrogen for 5 min followed by AIBN (300 mg, 1.8 mmol). The vials were sealed and vortexed again before polymerization was allowed to proceed for 48 h in a water bath at 55 °C. The resulting bulk polymers were released as monoliths by careful crushing of the glass vials, after which they were finely ground and sieved to achieve particles less than 77 µm in diameter. To prevent static charging, a few drops of methanol were added while grinding. Particles were subsequently suspended in a mixture of methanol (50 mL) and water (100 mL); then, they were subjected to centrifugation (150× *g*, 20 °C, 5 min) using polypropylene tubes, and the supernatant was discarded. Washing with water (6 × 150 mL) followed by acetone (1 × 25 mL) was repeated a total of seven times. Superfine particles were finally removed by suspension in acetone (6 × 50 mL), which was allowed to settle for 2 min every time. MIP and NIP (non-imprinted mock polymers) were dried by vacuum filtration. The successful release of RHO during washing was monitored by visual inspection and by precipitation with aqueous barium chloride (0.25 g/mL; a reddish precipitate insoluble in hydrochloric acid (0.1 M) is obtained when a drop of washing water is placed on a filter paper followed by a drop of the barium chloride solution [40]). For routine MIP production, visual monitoring of the wash-out process was considered sufficient, as the possibly remaining RHO does not interfere with CIT determination. However, precipitate formation provides additional proof that RHO deterioration as a result of the applied reaction conditions can be excluded.

### 4.4. Core-Shell Polymer Synthesis

The preparation of core-shell polymers followed previously published polymerization protocols [29,31,41,42,43]. Briefly, synthesis was carried out in three steps: core synthesis, core enlargement, and shell formation. To form the core first, EGDMA (1.895 mL, 10 mmol) and DAEM (0.507 mL, 3 mmol) were dissolved in a mixture of acetone (3.75 mL) and methanol (11.25 mL) placed in a headspace vial (50 mL). Following sonication for 15 min, the mixture was cooled to 0 °C for 1 h, AIBN (164 mg, 1 mmol) was added, and the mixture was flushed with nitrogen for 10 min. Subsequent polymerization was carried out at 55 °C for 5 h using a water bath while stirring at 500 rpm. Following centrifugation (133× *g*, 20 °C, 1 min) and discarding of the supernatant, core enlargement was carried out using a freshly prepared porogen/cross-linker/monomer/initiator mixture added to the core particles. Final shell formation was achieved using DAEM (1.014 mL, 6 mmol) and RHO (412 mg, 2 mmol), which were premixed in a separate vial containing acetone (6.25 mL) and methanol (18.75 mL), sonicated for 15 min, and cooled down to 0 °C for 1 h. EGDMA (1.895 mL, 10 mmol) was added, and the whole mixture was transferred to the headspace vial containing the previously prepared polymer cores obtained by centrifugation (133× *g*, 20 °C, 1 min). After sonication for 5 min, AIBN (164 mg, 1 mmol) was added, and the vial was flushed with nitrogen for 10 min followed by polymerization as described above. The resulting core-shell particles were washed by suspension/centrifugation (133× *g*, 20 °C, 5 min) using polypropylene tubes as shown in Table 7 and finally dried by vacuum filtration (see Supplementary Materials for further details).

### 4.5. Cartridge Packing

Bulk or core-shell polymers were packed for MISPE using the slurry method. Polymer particles (150 ± 1 mg) were placed into an empty polypropylene SPE cartridge equipped with a bottom frit and suspended in 2 mL of methanol. The solvent was removed by vacuum filtration, and a second frit was stacked onto the swollen polymer. Cartridges were either directly used for MISPE or dried and stored at room temperature protected from light (polymer particles will shrink to their original size but will recover when moistened again).

### 4.6. Extraction and MISPE

Cereal products (40 g) were finely ground, suspended in methanol/water (70/30, *v*/*v*, 200 mL), and shaken horizontally for 1 h at 200 rpm using polypropylene tubes. Centrifugation (3774× *g*, 20 °C, 10 min) followed by microfiltration (PTFE, 0.45 µm) provided a supernatant suitable for immediate analysis. If required, spiking with CIT (400 ng/mL) or OTA (30 ng/mL) was carried out afterwards to exclude any influence by extraction recovery. MISPE cartridges were flushed with a mixture of 2% ammonia (25% aq.) in methanol (3 mL) followed by conditioning with methanol/water (70/30, *v*/*v*, 3 mL). Samples (1 mL) were subsequently loaded onto the columns followed by washing with 1% acetic acid in methanol (0.8 mL). CIT and OTA were eluted with 2% (25% aq.) ammonia in methanol (2 mL, 2 × 0.8 mL with backflushing for core-shell polymers) at a flow rate of 2 mL/min (approximately 1 drop/2 s), collected in amber vials and evaporated to dryness using a vacuum concentrator (RVC 2-18, Christ, Osterode, Germany) at 50 °C (40 °C for core-shell polymers). Extracts were taken up in 10 mM orthophosphoric acid/acetonitrile (80/20, *v*/*v*, 1 mL) or methanol/water (50/50, *v*/*v*, 1 mL; core-shell polymers), filtered (PTFE, 0.45 µm), and subjected to LC analysis (20 µL).

### 4.7. Immunoaffinity Chromatography

IAC was carried out following the supplier’s instructions [13]. Briefly, samples (2 mL) were diluted with PBS to a final volume of 20 mL, of which 10 mL were loaded onto the IAC columns at a flow rate of approximately 2 mL/min. The columns were washed with 0.1% Tween 20 (10 mL in 10 mM orthophosphoric acid) followed by 10 mM orthophosphoric acid (10 mL, pH 2.5). CIT was eluted using methanol (1 mL) followed by water (1 mL) into amber vials and subjected to LC analysis (40 µL).

### 4.8. Quantification of Citrinin and Ochratoxin A

Liquid chromatography of CIT and OTA was carried out using an Agilent (Waldbronn, Germany) 1200 LC system equipped with a fluorescence detector (λ_ex_ = 335 nm, λ_em_ = 480 nm). Chromatographic separation was achieved on a Phenomenex Luna C_18_ column (4.6 × 100 mm, 3 µm; Aschaffenburg, Germany) using gradient elution (A: 10 mM orthophosphoric acid, B: acetonitrile; min/% B: 0/20, 6/60, 9/30, 12/20) at 40 °C and a flow rate of 1 mL/min. Following DIN 32645 [34], limits of detection (LOD) and quantification (LOQ) were determined using three-fold matrix-matched, equidistant five-point calibration curves within the expected LOQ range (Table 8). Recovery and precision data for each matrix were subsequently obtained at the determined LOQ (*n* = 6).

## Figures and Tables

**Table 1:** Surrogates selected for imprinting as identified by a pharmacophore search.

Surrogate	Pharmacophore Fit Score (%)
1,3-dihydroxy-2-naphthoic acid	66.1
rhodizonic acid dihydrate	65.9
1,4-dihydroxy-2-naphthoic acid	65.7
1-hydroxy-2-naphthoic acid	65.6
2-oxocyclohexane carboxylic acid	56.2
2-naphthoic acid	56.0
salicylic acid	55.9

**Table 2:** LOD and LOQ derived for CIT quantification by MISPE using bulk MIP imprinted by RHO (following DIN 32645 [34] using a matrix-matched five point calibration, *n* = 3).

Matrix	LOD (mg/kg)	LOQ (mg/kg)
rice	0.01	0.03
rice crispies	0.02	0.08
wheat flour	0.004	0.02
pasta	0.003	0.01
oats	0.1	0.5

**Table 3:** Recovery, precision, and reusability for CIT quantification by MISPE using bulk MIP imprinted by RHO (recovery as mean ± RSD, *n* = 6).

Matrix	LOQ (mg/kg)	Recovery (%)			
		MISPE			IAC
		1 ^a^	2 ^a^	3 ^a^	
rice	0.03	62.0 ± 4.8	75.9 ± 5.5	109.0 ± 3.6	-
rice crispies	0.08	88.0 ± 3.8	78.4 ± 1.5	94.7 ± 18.2	41.5 ± 74.8
wheat flour	0.02	71.0 ± 20.4	65.5 ± 7.1	137.2 ± 10.1	-
pasta	0.01	55.0 ± 13.3	107.2 ± 7.1	144.2 ± 2.3	57.7 ± 29.9
oats	0.5	96.5 ± 1.9	96.2 ± 0.9	89.3 ± 1.6	41.6 ± 74.7

^a^ indicating cycle 1–3 of repeated usage.

**Table 4:** CIT (400 ng/mL, 2 mg/kg) recovery by MISPE using RHO-derived core-shell and type II bulk MIP and NIP (mean ± RSD, *n* = 6).

Matrix	Polymer	Recovery (%)
methanol/water (70/30, *v*/*v*)	core-shell MIP	97.5 ± 2.2
	core-shell NIP	105.5 ± 1.8
	bulk MIP (type II)	103.2 ± 2.3
	bulk NIP (type II)	103.0 ± 1.4
oats	core-shell MIP	97.0 ± 2.9
	core-shell NIP	96.9 ± 0.3
	bulk MIP (type II)	92.0 ± 1.8
	bulk NIP (type II)	90.0 ± 1.6

**Table 5:** LOD and LOQ derived for CIT quantification by MISPE using core-shell MIP imprinted by RHO (matrix-matched five-point calibration following DIN 32645, recovery as mean ± RSD, *n* = 3).

Matrix	Type	LOD (mg/kg)	LOQ (mg/kg)	Recovery (%)
pasta	MIP	0.014	0.048	96.0 ± 1.3
	NIP	0.011	0.037	97.9 ± 3.4
oats	MIP	0.12	0.45	85.1 ± 1.8
	NIP	0.15	0.55	97.6 ± 1.1

**Table 6:** Recovery and precision of OTA (30 ng/mL) quantification by MISPE using core-shell MIP and NIP imprinted by RHO (mean ± RSD, *n* = 6).

Matrix	Type	Recovery (%)
methanol/water (70/30, *v*/*v*)	MIP	82.7 ± 5.0
	NIP	95.8 ± 7.3
oats	MIP	76.8 ± 3.9
	NIP	88.6 ± 1.2

**Table 7:** Washing steps for core-shell MIP and NIP production.

1.	2.	3.	4.	5.
methanol	water	methanol	water	acetone
1 × 50 mL	5 × 50 mL	5 × 50 mL	10 × 50 mL	1 × 50 mL

**Table 8:** Matrix-matched calibration levels for bulk and core-shell MIP and NIP.

Polymer Type	Matrix	Calibration Range (mg/kg)
bulk MIP	rice	0.025, 0.050, 0.075, 0.100, 0.125
	rice crispies	0.05, 0.10, 0.15, 0.20, 0.25
	wheat flour	0.025, 0.050, 0.075, 0.100, 0.125
	pasta	0.025, 0.050, 0.075, 0.100, 0.125
	oats	0.5, 1.0, 1.5, 2.0, 2.5
core-shell MIP/NIP	pasta	0.025, 0.050, 0.075, 0.100, 0.125
	oats	0.5, 1.0, 1.5, 2.0, 2.5

## Data Availability

The data presented in this study are available in Supplementary Materials.

## Supplementary Materials

The following are available online. Figure S1: CIT short-term stability. Figure S2: CIT and OTA long-term stability. Table S1: Optimization of MISPE elution using bulk polymers imprinted by 1,4-dihydroxy-2-naphthoic acid. Table S2: Optimization of MISPE washing as estimated by recovery and FLD noise. Table S3: Optimization of washing volume. Table S4: Different combinations of porogen, cross-linker, and functional monomers and their effect on the size, form, and behavior of core-shell MIP imprinted by RHO.

## Sample Availability

Samples of the MIP are available from the authors.

## Appendix A

**Table A1:** CIT (400 ng/mL, 2 mg/kg) recoveries achieved by clean-up of rice, rice crispies, wheat flour, pasta, and oats applying MISPE, IAC, and SPE. Polymers for MISPE were imprinted using surrogates identified by pharmacophore modeling (mean ± RSD, *n* = 6).

Clean-Up	Imprinting Surrogate	Recovery (%)				
		Rice	Rice Crispies	Wheat Flour	Pasta	Oats
MISPE	2-naphthoic acid	49.5 ± 6.6	71.1 ± 53.6	32.0 ± 2.2	66.4 ± 13.9	90.7 ± 2.1
	1-hydroxy-2-naphthoic acid	60.8 ± 15.9	119.1 ± 6.0	62.4 ± 20.9	72.8 ± 4.3	76.4 ± 5.0
	1,3-dihydroxy-2-naphthoic acid	72.9 ± 4.4	59.2 ± 12.2	38.3 ± 15.0	99.8 ± 6.1	79.8 ± 2.5
	1,4-dihydroxy-2-naphthoic acid	88.0 ± 1.7	88.7 ± 34.8	67.8 ± 17.2	125.9 ± 0.3	94.0 ± 3.8
	salicylic acid	54.5 ± 4.4	120.8 ± 3.4	36.5 ± 13.3	120.8 ± 1.3	87.8 ± 0.9
	2-oxocyclohexane carboxylic acid	51.6 ± 12.0	116.6 ± 2.7	30.2 ± 10.6	114.8 ± 2.9	77.2 ± 5.8
	rhodizonic acid dihydrate	94.0 ± 1.2	118.4 ± 4.0	111.7 ± 2.6	127.4 ± 2.6	114.0 ± 0.8
	non-imprinted	61.4 ± 6.5	126.5 ± 1.1	21.1 ± 18.8	91.5 ± 24.4	78.6 ± 3.4
IAC	–	97.9 ± 9.4	121.4 ± 2.4	87.1 ± 2.3	114.3 ± 10.5	96.5 ± 8.9
SPE (C_18_)	–	50.9 ± 4.6	72.8 ± 17.9	45.3 ± 7.5	50.5 ± 13.8	39.4 ± 22.7
