# Supplementary material for: Guiding Molecularly Imprinted Polymer Design by Pharmacophore Modeling
Source: Molecules. 2021 Aug 23;26(16):5101. doi: 10.3390/molecules26165101 (PMC8402217; doi:10.3390/molecules26165101)
Supplement: Supplementary file 1 [file molecules-26-05101-s001.zip › molecules-1334501-supplementary.pdf]

Supplementary Information

Guiding molecularly imprinted polymer design by pharmacophore modeling

Wiebke Derz, Melita Fleischmann and Paul W. Elsinghorst

1. MISPE Development

1.1 Bulk Polymers

Initial MISPE experiments used bulk polymers imprinted by 1,4-dihydroxy-2-naphthoic acid following a published MISPE protocol for CIT [1]. As CIT could not be recovered under these conditions, first adaptations focused on elution as well as the total amount of polymer per cartridge. As shown in **Table S1**, switching from acidic to basic elution conditions (1–3% ammonia in methanol) provided acceptable recoveries, which could be further improved by a 25% reduction in polymer per cartridge.

Table S1: MISPE elution optimization using bulk polymers imprinted by 1,4-dihydroxy-2-naphthoic acid and 1 mL CIT (400 ng/mL in methanol/water, 70/30, *v*/*v*) (n = 6).

ammonia in methanol (%)	polymer/cartridge (mg)	recovery (%)
1	200	81.2 ± 2.1
2	200	85.0 ± 3.3
3	200	84.8 ± 3.8
2	150	92.2 ± 2.2

Applying these modifications rice, rice crispies, wheat flour, pasta and oat matrix extracts were spiked to 2 mg/kg CIT and subjected to MISPE using MIP and NIP obtained from different surrogate templates (see also **Table A1**). The most promising surrogate from this screening (RHO) was subsequently used to optimize the washing step. Therefore, matrix extracts were subjected to MISPE using MIP imprinted by RHO and washed with different aqueous and organic solvents. Results were evaluated with respect to overall recovery and matrix removal as estimated by FLD noise in the range preceding CIT (**Table S2**). The most efficient clean-up was achieved when using methanol containing 1% acetic acid, although recoveries were lower than obtained when washing with water.

Table S2: MISPE washing optimization using spiked matrix extracts (400 ng/mL CIT), different aqueous and organic solvents and MIP imprinted by RHO (recovery as mean ± RSD, noise as mean ± SD, noise range: 6.15–7.35 min, n = 6).

washing solvent	matrix	recovery (%)	noise (LU)
water	rice	84.9 ± 1.7	0.75 ± 0.01
	rice crispies	90.9 ± 2.8	1.1 ± 0.1
	wheat flour	76.7 ± 12.3	0.031 ± 0.015
	pasta	92.7 ± 3.3	0.19 ± 0.01
	oats	84.0 ± 8.0	12.6 ± 0.8

washing solvent	matrix	recovery (%)	noise (LU)
0.1% Tween 20 aq. followed by water	rice	67.4 ± 5.7	0.67 ± 0.04
	rice crispies	91.3 ± 5.7	1.6 ± 0.1
	wheat flour	89.4 ± 6.8	0.037 ± 0.008
	pasta	99.3 ± 1.2	0.19 ± 0.01
	oats	97.4 ± 1.1	13.4 ± 0.3
0.1% Tween 20 in 10 mM orthophosphoric acid followed by water	rice	74.4 ± 3.1	0.78 ± 0.01
	rice crispies	100.1 ± 3.6	1.4 ± 0.1
	wheat flour	77.7 ± 19.9	0.034 ± 0.003
	pasta	99.2 ± 1.1	0.18 ± 0.01
	oats	109.6 ± 0.9	11.0 ± 0.9
methanol/water (1/1, *v*/*v*)	rice	67.6 ± 1.4	0.75 ± 0.02
	rice crispies	73.4 ± 4.3	0.72 ± 0.08
	wheat flour	54.8 ± 25.6	0.022 ± 0.004
	pasta	95.6 ± 2.6	0.19 ± 0.01
	oats	89.4 ± 4.7	11.1 ± 0.9
methanol/water (1/1, *v*/*v*), 1% acetic acid	rice	85.2 ± 5.2	0.76 ± 0.04
	rice crispies	89.7 ± 0.4	0.92 ± 0.06
	wheat flour	104.0 ± 6.0	0.025 ± 0.001
	pasta	102.8 ± 1.9	0.19 ± 0.01
	oats	108.5 ± 2.9	13.4 ± 1.0
methanol	rice	65.4 ± 5.0	0.30 ± 0.06
	rice crispies	71.9 ± 2.9	0.40 ± 0.02
	wheat flour	40.3 ± 20.7	0.023 ± 0.005
	pasta	84.7 ± 3.0	0.10 ± 0.01
	oats	101.6 ± 1.3	3.0 ± 0.5
methanol, 1% acetic acid	rice	57.4 ± 17.3	0.17 ± 0.04
	rice crispies	84.8 ± 3.7	0.38 ± 0.06
	wheat flour	53.8 ± 11.7	0.018 ± 0.002
	pasta	82.5 ± 1.9	0.06 ± 0.01
	oats	107.4 ± 1.1	3.5 ± 0.9

In addition, different volumes of washing solvent were evaluated considering acidic methanol and water for comparison (**Table S3**). Oats as the most complex matrix (extracts spiked to 400 ng/mL CIT) were used for an initial screening in the range of 0.4–1.0 mL (for acidic methanol this range was extended to 2.0 mL, which however resulted already in a loss of analyte). As 1.0 mL instead of 0.8 mL acidic methanol did not provide less noise, but a slight loss in recovery, a final 0.8 mL wash step was applied for clean-up of the remaining matrices. As shown in **Table S3**, washing with 1% acetic acid in methanol again provided lower noise while recoveries remained around 90%.

Table S3: Wash volume optimization using spiked matrix extracts (400 ng/mL CIT) and MIP imprinted by RHO (recovery as mean ± RSD, noise as mean ± SD, noise range: 6.15–7.35 min, n = 6).

matrix	washing solvent	volume (mL)	recovery (%)	noise (LU)
oats	water	0.4	87.1 ± 4.0	5.2 ± 0.5
		0.6	89.4 ± 2.6	5.4 ± 0.3
		0.8	92.8 ± 3.5	4.9 ± 0.6
		1.0	87.9 ± 2.3	5.3 ± 0.4
oats	methanol, 1% acetic acid	0.4	92.7 ± 0.9	2.6 ± 0.2
		0.6	91.7 ± 2.0	1.8 ± 0.1
		0.8	93.5 ± 1.4	1.8 ± 0.2
		1.0	90.8 ± 1.0	1.1 ± 0.3
		2.0	81.3 ± 1.1	0.16 ± 0.016
rice	water	0.8	78.4 ± 2.5	0.070 ± 0.011
	methanol, 1% acetic acid	0.8	91.4 ± 1.7	0.036 ± 0.011
rice crispies	water	0.8	79.2 ± 1.6	0.52 ± 0.02
	methanol, 1% acetic acid	0.8	92.7 ± 0.6	0.48 ± 0.13
wheat flour	water	0.8	84.3 ± 1.5	0.099 ± 0.001
	methanol, 1% acetic acid	0.8	89.8 ± 1.4	0.053 ± 0.005
pasta	water	0.8	86.0 ± 5.1	0.27 ± 0.006
	methanol, 1% acetic acid	0.8	91.6 ± 0.4	0.12 ± 0.007

1.2 Core-shell Polymers

To identify optimal polymerization conditions, different compositions of porogen (methanol, acetonitrile), cross-linker (TRIM, EGDMA) and functional monomers (DAEM, MAA, MAM) were used for core-shell polymer synthesis. The obtained polymers were subsequently analyzed for their size and general appearance using light microscopy (**Table S4**). In contrast to bulk polymerization, TRIM proved unsuitable as a cross-linker for core-shell polymerization as the obtained particles were too small for MISPE showing strong agglomeration. EGDMA as a cross-linker in combination with the functional monomer DAEM provided microspheres of narrow size dispersion, while methanol used as the porogen and to solubilize RHO led to improved particle formation.

Table S4: Compositions of porogen (methanol, acetonitrile), cross-linker (trimethylolpropane trimethacrylate, TRIM; ethylene glycol dimethacrylate, EGDMA) and functional monomers (*N,N*-dimethylaminoethyl methacrylate, DAEM; methacrylic acid, MAA; methacrylamide, MAM) for core-shell MIP imprinted by RHO. Microscopy (200× magnification) was used to evaluate their sutability for MISPE cartridge packing in terms of size, form and behavior.

porogen	cross-linker	functional monomer	description/microscopy	
acetonitrile	EGDMA	DAEM	- extremely fine particles<br>- fluffy texture<br>- bad handling	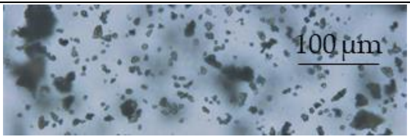
methanol	TRIM	MAA	- uniformly sized particles<br>- Ø approx. 20 µm<br>- no agglomeration	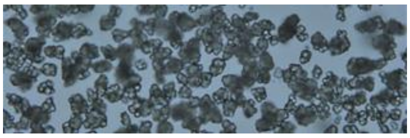

porogen	cross-linker	functional monomer	description/microscopy	
methanol	TRIM	MAM	- very fine particles,<br>- strong agglomeration	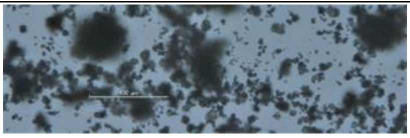
methanol	EGDMA	DAEM	- uniformly sized particles<br>- Ø approx. 20 µm<br>- no agglomeration	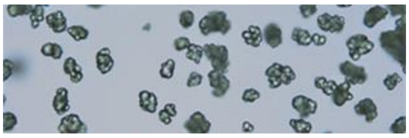
methanol	EGDMA	MAA	- uniformly sized particles<br>- Ø approx. 10 µm<br>- agglomeration	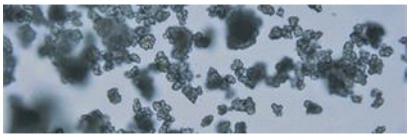
methanol	EGDMA	MAM	- uniformly sized particles<br>- Ø approx. 5 µm<br>- strong agglomeration	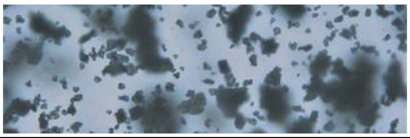

2. Citrinin stability

As research progressed doubts arose about the stability of CIT in those media used for MISPE and LC analysis (bulk polymers: 10 mM orthophosphoric acid/acetonitrile (80/20, *v/v*); core-shell polymers: methanol/water (50/50, *v/v*)). Neumann trend analysis of short-term stability experiments carried out over 12 hours revealed a significant degradation of CIT when stored in 10 mM orthophosphoric acid/acetonitrile (80/20, *v/v*), whereas storage in methanol/water (1/1, *v/v*) did not have a negative effect (**Figure S1**).

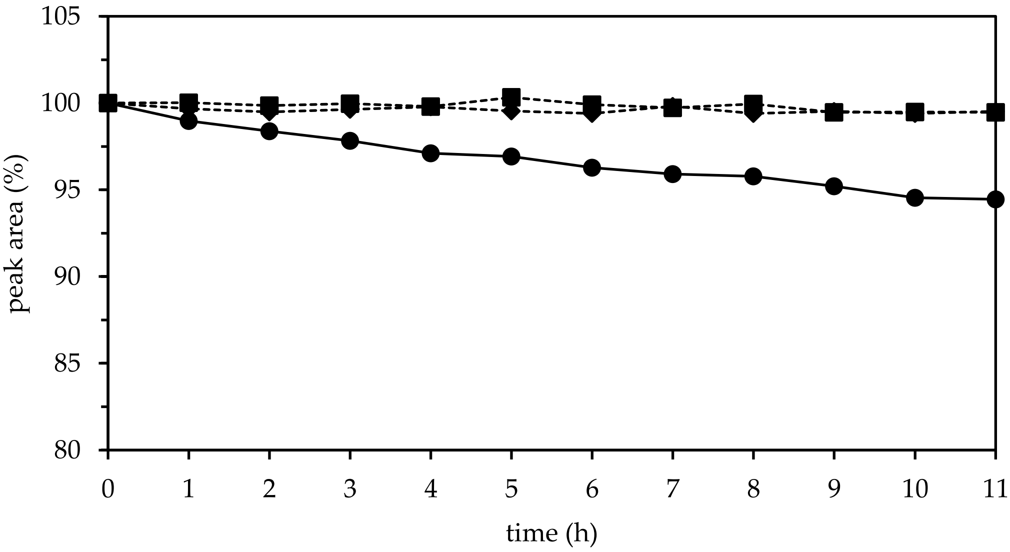

Figure S1: Short-term stability of CIT (●: 10 mM orthophosphoric acid/acetonitrile (80/20, *v/v*), -18 °C; ■: methanol/water (1/1, *v/v*), -18 °C; ♦: methanol/water (1/1, *v/v*), +4 °C).

Additional long-term stability tests of CIT/OTA mixtures using the LC thermostatic sampler (+18 °C) corroborated the instability of CIT in 10 mM orthophosphoric acid/acetonitrile (80/20, *v/v*) whereas OTA remained stable (**Figure S2**). Switching from bulk to core-shell polymers was therefore accompanied by a switch in reconstitution solution to methanol/water (1/1, *v/v*) and a lower evaporation temperature (40 °C).

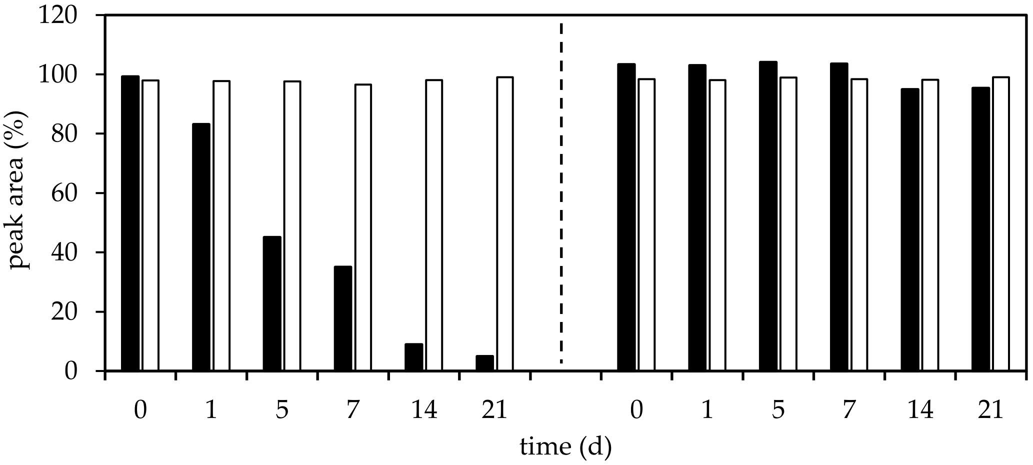

Figure S2: Long-term stability of CIT (■) and OTA (□) (left: 10 mM orthophosphoric acid/ acetonitrile (80/20, *v/v*); right: methanol/water (1/1, *v/v*)).

References

1. Appell, M.; Jackson, M.A.; Wang, L.C.; Bosma, W.B. Determination of Citrinin Using Molecularly Imprinted Solid Phase Extraction Purification, HPLC Separation, and Fluorescence Detection. *J. Liq. Chromatogr. R. T.* **2015**, *38*, 1815–1819, doi:10.1080/10826076.2015.1113546.
